# Global Challenges for Cancer Imaging

**DOI:** 10.1200/JGO.17.00036

**Published:** 2017-09-08

**Authors:** Heinz-Peter Schlemmer, Leonardo K. Bittencourt, Melvin D’Anastasi, Romeu Domingues, Pek-Lan Khong, Zarina Lockhat, Ada Muellner, Maximilian F. Reiser, Richard L. Schilsky, Hedvig Hricak

**Affiliations:** **Heinz-Peter Schlemmer**, German Cancer Research Center, Heidelberg; **Melvin D’Anastasi** and **Maximilian F. Reiser**, Ludwig-Maximilians-University Hospital, Munich, Germany; **Leonardo K. Bittencourt**, Fluminense Federal University, Niterói; **Leonardo K. Bittencourt** and **Romeu Domingues**, Clínica de Diagnóstico por Imagem (CDPI/Dasa), Rio de Janeiro, Brazil; **Pek-Lan Khong**, University of Hong Kong, Queen Mary Hospital, Hong Kong, China; **Zarina Lockhat**, University of Pretoria, Steve Biko Academic Hospital, Pretoria, South Africa; **Ada Muellner** and **Hedvig Hricak**, Memorial Sloan Kettering Cancer Center, New York, NY; and **Richard L. Schilsky**, American Society of Clinical Oncology, Alexandria, VA.

## Abstract

Imaging plays many essential roles in nearly all aspects of high-quality cancer care. However, challenges to the delivery of optimal cancer imaging in both developing and advanced countries are manifold. Developing countries typically face dramatic shortages of both imaging equipment and general radiologists, and efforts to improve cancer imaging in these countries are often complicated by poor infrastructure, cultural barriers, and other obstacles. In advanced countries, on the other hand, although imaging equipment and general radiologists are typically accessible, the complexity of oncologic imaging and the need for subspecialists in the field are largely unrecognized; as a result, training opportunities are lacking, and there is a shortage of radiologists with the necessary subspecialty expertise to provide optimal cancer care and participate in advanced clinical research. This article is intended to raise awareness of these challenges and catalyze further efforts to address them. Some promising strategies and ongoing efforts are reviewed, and some specific actions are proposed.

## INTRODUCTION

Imaging plays many essential roles in nearly all aspects of high-quality cancer care, from diagnosis through treatment and follow-up. Cross-sectional imaging technologies now exist that can accurately detect cancer, assess its spread, and, in some cases, provide information about its biology. However, vast inequities in access to imaging equipment and expertise exist between developing and developed countries. Furthermore, even in developed countries, the complexity of oncologic imaging is underappreciated, and the supply of imagers with the specialized training necessary to provide optimal cancer care is far from adequate. This article, which was inspired by discussions held at and after the 2014 meeting of the International Cancer Imaging Society, is intended to increase awareness of these challenges and catalyze further efforts to overcome them. Some promising strategies and ongoing efforts to advance cancer imaging are reviewed, and some specific actions are proposed.

## INEQUITIES IN PATIENT OUTCOMES FOR PATIENTS WITH CANCER, HEALTH CARE SPENDING, AND IMAGING AVAILABILITY

Cancer is the world’s leading cause of death, and it is expected that by 2030, the number of new cancer cases per year, which was estimated at 14 million in 2012, will have increased by more than 50%, to 22 million.^1^ Both the majority of cancer cases and the majority of cancer deaths now occur in developing nations, where resources for coping with cancer are least available.^2^

Although the direct effects of imaging on long-term patient outcomes have not been studied extensively and are difficult to isolate and quantify, in regions with better cancer outcomes, imaging is considered a cornerstone of cancer care. A study conducted in Europe showed not only that all-cancer relative survival was better in countries with higher overall health care expenditures and greater numbers of computed tomography (CT) and magnetic resonance imaging (MRI) units per capita but also that it correlated directly with the number of MRI units per capita—a finding the authors described as “consistent with the known importance of early and accurate diagnosis in cancer survival.”^3(p85)^ Imaging allows cancers to be detected when they are smaller and more likely to be curable. For example, growing use of mammography has lowered the stage of breast cancers at diagnosis and, together with advances in treatment, has contributed to increases in breast cancer survival observed in Europe, the United States, Australia, South Korea, Japan, and Singapore.^4-7^ The use of imaging for staging can often replace more invasive staging procedures and is critical for determining the most appropriate treatment approach and enabling minimally invasive treatments. Furthermore, imaging is used routinely for assessing responses to numerous cancer therapies and determining the effectiveness and duration of treatment.

Defining the optimal numbers of CT, MRI, or other types of imaging units per million population would be a complex task and would need to be done on a country-by-country or even more localized basis. It is clear that easy access to imaging equipment can lead to its overuse—a problem that is exacerbated when the physicians who refer patients for imaging profit from its use.^8^ However, it is also clear that a lack of imaging equipment can lead to long wait times for imaging examinations and create geographic barriers to access.^9^ Unfortunately, inequities in the distribution of imaging equipment between economically advanced and developing countries are pronounced. For example, the overall numbers of CT and MRI units per million inhabitants are 24.1 and 18.5, respectively, in Western Europe but just 13 and 6.5, respectively, in Central and Eastern Europe.^10^ According to a report published in 2015, the numbers of CT units per million population among the 32 Organisation for Economic Co-operation and Development countries ranged from 5.3 in Mexico to 101.3 in Japan at last count.^9^

Within many developing countries—as well as some economically advanced ones— inequities in the availability, accessibility, and affordability of imaging facilities among different geographic regions and sectors of the population are also striking. In China in 2009, for example, Shanghai had 3.2 MRI scanners per million population, whereas the rural province of Hunan had just 1.3.^11^ Although China is estimated to have doubled its high-end imaging technology resources from 2008 to 2012, at the end of that period, the number of MRI scanners per unit population in Shanghai remained twice that in Hunan.^12^

Highly uneven distribution of health care resources between the public and private sectors is also a common problem. For example, in Brazil, public health care facilities tend to the needs of approximately 75% of the population but possess only approximately 16% of CT scanners and 6% of MRI scanners.^13^ Similarly, in South Africa, > 90% of the MRI scanners—as well as the majority of radiologists—are located in the private sector, which cares for only approximately 16% of the population.^14^

## GAPS IN IMAGING EXPERTISE IN HIGHER-INCOME COUNTRIES

With the increasing demand for cancer imaging worldwide, there is a growing need for expertise in the performance and reporting of oncology examinations. It is not surprising that in low-income countries, where general radiologists are in short supply, cancer imaging expertise is scarce. However, there is little awareness that even in economically advanced countries such as the United States, where imaging services are widely available and a large share of the examinations performed by radiology practices are oncologic imaging studies, the shortage of trained oncologic imagers is an obstacle to the delivery, as well as the advancement, of high-quality cancer care. This section discusses the need to foster specialized cancer imaging expertise to optimize cancer care in higher-income countries with sound health care infrastructures.

High-quality imaging requires multifaceted, disease-specific knowledge and sophisticated communication skills. It entails effective planning and performance of the examination itself, image postprocessing, image interpretation, and clinically relevant, standardized reporting. Not only does the oncologic radiologist need to be familiar with a variety of tumor entities, their patterns of spread, and their appearances on various imaging modalities, but he/she also needs to be able to assess tumor response to^15^ and complications from different therapies^16^ as well as the varying patterns of tumor recurrence. In addition, he/she must understand the practices and needs of the various clinical partners (eg, from surgery, radiation oncology, and medical oncology) involved in cancer care.

Research has consistently shown that outcomes are better for patients with cancer when they are treated at dedicated tertiary care cancer centers rather than in less-specialized settings. For example, one recent analysis found that the risk-adjusted probability of death at 1 year was 10 percentage points lower for patients treated at freestanding specialty cancer hospitals than for patients with cancer treated at community hospitals in the United States.^17^ At many dedicated tertiary care cancer centers and large hospitals known for providing high-quality cancer care, the optimal management of patients with cancer is discussed at tumor conferences comprising specialized multidisciplinary teams, of which radiologists are integral members. Radiologist members of these teams have subspecialized expertise and are often asked to perform second-opinion reporting of oncologic imaging studies obtained at other facilities. Second-opinion reporting by subspecialized radiologists has been shown to improve diagnostic accuracy and affect patient care.^18-20^ In a study by Lakhman et al,^21^ for instance, two gynecologic oncologic surgeons retrospectively compared 469 consecutive second-opinion MRI interpretations rendered by gynecologic-oncologic radiologists to the initial outside reports; for each surgeon, it was found that second-opinion review of gynecologic MRIs would have affected the management of at least 20% of patients and would have changed patient management from a surgical to a nonsurgical approach for approximately 7% of patients. Comparison with histopathology or minimum 6-month imaging follow-up showed that second-opinion interpretations were correct in 103 (83%) of 124 cases with clinically relevant discrepancies between initial and second-opinion reports.^21^ Table 1 summarizes the results of this and other recent studies comparing initial oncologic imaging readings with second-opinion readings performed at comprehensive cancer centers; as shown, the reported levels of disagreement between initial and second-opinion readings range from 13% to 56%, whereas the reported percentages of cases for which second-opinion readings indicated the need for a change in management range from 13% to 53.5%.^19,21-29^ Requests for second-opinion reporting are continually increasing and constitute a considerable part of the subspecialized radiologist’s workload in larger centers. However, although it is essential to the overall quality of oncologic imaging services, second-opinion reporting is currently not widely reimbursed outside of the United States.

**Table 1:**
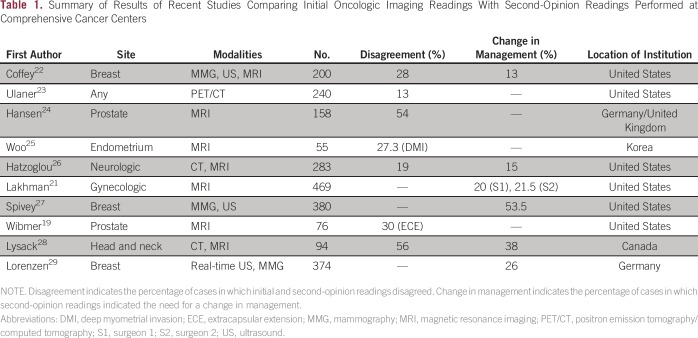
Summary of Results of Recent Studies Comparing Initial Oncologic Imaging Readings With Second-Opinion Readings Performed at Comprehensive Cancer Centers

In economically advanced countries, the need for specialization in (and within) oncologic imaging is being exacerbated by the increasing use of molecularly targeted treatments and immunotherapy, for which response assessment on imaging is often complex. Because the mechanisms of action of most targeted treatments differ from those of traditional cytotoxic chemotherapies, a variety of new imaging-based response assessment criteria have been developed, and more are needed.^15^ Examples of response assessment criteria developed as a result of newly available therapies in recent years include the Choi criteria for gastrointestinal stromal tumors and the Immune-Related Response Criteria, as well as the recently published modified Response Evaluation Criteria in Solid Tumors 1.1 for immune-based therapeutics.^30-32^ Disease-specific criteria include the Lugano classification for lymphomas, recently published provisional recommendations as a refinement of the latter for patients receiving immunomodulatory therapies,^33,34^ and the just-published proposed new response evaluation criteria in lymphoma (RECIL 2017).^35^ Given that 10 new anticancer drugs received regulatory approval in the United States in 2014 alone, and hundreds more are in development,^36^ it is easy to appreciate why specialized knowledge of the various tumor response assessment criteria is important for radiologists reporting in clinical trials.

Radiologists who have completed fellowships in body imaging, chest imaging, or other areas can acquire subspecialized expertise in imaging certain kinds of cancer by working in centers that provide team-based, multidisciplinary cancer care. In addition, some dedicated tertiary care cancer centers offer fellowships specifically in oncologic imaging subjects; these fellowships allow radiologists to develop subspecialty expertise more rapidly and may also give them valuable experience in cutting-edge research. Subspecialization itself should furthermore ensure that cutting-edge knowledge in oncologic imaging is maintained and advanced through ongoing training and close and sustainable collaboration with a multidisciplinary clinical oncology team.

Unfortunately, even in high-income countries such as Japan, Korea, Hong Kong, and the United States, oncologic imaging is still not a formally recognized subspecialty for which certification is available. In the United States, out of more than 200 imaging fellowships offered, only a handful are formal oncologic imaging fellowships; furthermore, the supply of radiologists with any kind of fellowship training is limited, and many imaging facilities do not require it.

In the future, advanced computer tools for analyzing images (discussed more extensively in the next section of this article) may help raise the quality of oncologic imaging among general radiologists as well as subspecialists. However, in the nearer term, one way to help optimize cancer imaging in higher-income countries may be through the development of second-opinion image reporting services staffed and overseen by radiologists with subspecialty expertise in oncologic imaging. Some specialty cancer centers in the United States are already successfully offering such second-opinion services to patients being cared for at other institutions. However, to help reach all the patients who could potentially benefit from second-opinion reporting, many more such services would need to be established, and the supply of oncologic imaging subspecialists qualified to staff them would need to be expanded.

Professional societies must work together with government policymakers to disseminate awareness of the importance of specialized cancer imaging expertise. In addition, professional societies should formally recognize oncologic imaging as a subspecialty (or better yet, a set of subspecialties), develop and promote practice standards in oncologic imaging, develop training curricula and fund fellowships and other training opportunities in oncologic imaging, and encourage the development of second-opinion reporting services in oncologic imaging along with standards for their accreditation.

International efforts to advance some of these goals have already begun. For example, to promote quality standards and the recognition of oncologic imaging as a radiologic subspecialty, the European Society of Radiology (ESR) has included a chapter on oncologic imaging in both the European Training Curriculum for Radiology and the European Training Curriculum for Subspecialization in Radiology^37,38^; it is hoped that this will encourage national societies to also integrate oncologic imaging in their radiology training curricula. In 2000, the International Cancer Imaging Society was formed specifically to foster education in oncologic imaging, stimulate research, and bring together radiologists and clinicians involved in cancer care. Subsequently, the ESR founded the European Society of Oncologic Imaging. Opportunities for education in oncologic imaging—such as courses, workshops, and online webinars—are offered by societies such as the European Society of Oncologic Imaging, the International Cancer Imaging Society, and the Radiological Society of North America, to name just a few. Opportunities for more intensive, subspecialized training can also be accessed through international societies. For example, the European School of Radiology supplies applications to a number of short- and long-term visiting scholarships and fellowships at centers of excellence in Europe and the United States, which are funded by the ESR, national societies, the pharmaceutical industry, academic institutions, and other sources.

Within the last 6 years, European initiatives have also been launched to help standardize training and practice in hybrid imaging (eg, positron emission tomography–CT) and interventional radiology—two areas of imaging that are becoming increasingly important in cancer care.^39,40,41^ There is a need to increase collaborative efforts with societies that are not focused on imaging, such as the European Society for Medical Oncology or the European Society for Radiotherapy and Oncology, in the development of clinical practice guidelines as well as educational and research initiatives. Just as oncologic radiologists are key members of disease management teams, they need to be integrated into the leadership bodies that design broad, cancer-related policies and initiatives around the world.

## EFFORTS AND STRATEGIES FOR IMPROVING THE AVAILABILITY AND QUALITY OF ONCOLOGIC IMAGING IN LOWER-INCOME REGIONS

In low-resource regions, a lack of imaging equipment typically goes hand in hand with a paucity of radiologists, and those radiologists who are present are likely to face intense clinical workloads. Although in the developed world, the existing medical infrastructure enables medical subspecialization and networking between different medical facilities, the situation in the developing world demands that radiologists, especially those working in smaller and geographically isolated community hospitals, have a great breadth of expertise. Not only do they need to be able to exploit all the imaging methods available on site, they must also be able to examine all body parts and meet the requirements of the multidisciplinary therapy approaches available. Meeting these demands is, of course, challenging, and a variety of obstacles may complicate efforts to help.^42^ This section briefly considers technological innovations that may help to address the shortages of imaging equipment and radiologists in low-resources areas; it then reviews some promising efforts and strategies to address these shortages that would benefit from further engagement from the imaging and cancer care communities.

### How Can Communication Technology, Health Care Informatics, and Artificial Intelligence Help?

The growth of the Internet has already made teleradiology part of the daily experience of many radiologists. Oncologic radiologists are particularly familiar with teleradiology, because imaging studies acquired at other institutions are transmitted to their centers for second-opinion reporting and follow-up assessment. More extensive radiology consulting services can also be supplied remotely, including recommendation and implementation of state-of-the-art imaging protocols, quality control, image interpretation, and reporting of the imaging studies according to the most recent guidelines; in addition, clinical decision support can be supplied via oncologic radiologists’ participation in interdisciplinary tumor board sessions (eg, through videoconferencing).^43^

Cross-border telemedicine has huge potential to improve patient care in rural areas and developing countries by providing access to specialist services. By 2011, cross-border telemedicine programs had been attempted or were underway in at least 76 countries, according to a systematic review of the literature.^44^ Most of these were pilot programs between high- and low- or middle-income countries. The programs encountered a variety of obstacles, particularly with regard to funding, legal issues (eg, data security, liability), cultural factors (eg, language, lack of mutual trust), and contextual factors (eg, lack of reliable infrastructure for information exchange, lack of resources to implement recommendations made by remote physicians). Although many of the programs were found to be ineffective or unsustainable, those that fared best tended to rely on low-cost technologies and involve close twinning relationships with remote academic centers that provided training and mentorship of local personnel.^44^

The value of the global telemedicine market reached approximately $17.879 million in 2015 and was expected to increase at a compound annual growth rate of 18.7% from 2016 to 2022, according to one analysis.^45^ Technologies for data transfer and storage are continuously being developed, some of them specifically for low-resource settings.^46^ Further advances in health care informatics and the development of supraregional networks will help to overcome traditional barriers.^44^

Rapid developments in computerized clinical decision support and artificial intelligence (AI) could also be of particular value for advancing cancer care in low-resource regions. Decision support systems for selecting appropriate imaging methods and imaging protocols according to established guidelines are increasingly being used in the developed world and could potentially be adapted for many different settings. Clinical decision support for radiologists has also been developed to promote standardization of interpretation, terminology, and follow-up recommendations during the generation of imaging reports.^47^ Furthermore, various AI techniques, such as neuronal networks, fuzzy logic, evolutionary computation, deep learning, and computer vision are emerging, all of which are capable of solving clinical problems. Computer-aided detection and diagnosis (CAD) systems have evolved to semiautomatically or even automatically detect pulmonary nodules or breast lesions, among other pathologies.^48,49^ CAD systems and other postprocessing tools for image analysis hold great potential to facilitate image interpretation in oncology by aiding or automatically performing the detection, segmentation, measurement, and even characterization of suspicious lesions, as well as the quantification of changes during treatment. As suggested by Saurabh Jha and Eric Topol,^50^ the use of computers to detect and characterize abnormalities could free up radiologists to act as information specialists, who would interpret the data provided in its larger context, advise on the need for any further diagnostic testing, and integrate findings to guide treating physicians.^50^ If AI becomes sufficiently accurate and reliable for lesion detection and characterization, it could be used to screen populations faster than radiologists can and could eventually lead to significant cost savings; in turn, with the help of AI, a single information specialist could then “manage screening for an entire town in Africa,” Jha and Topol assert.^50(p2354)^

### Efforts and Strategies to Implement Change

As noted in “Public health oncology: a framework for progress in low- and middle-income countries,” success in advancing cancer care in such countries depends not just on the support of international partners but, even more importantly, on the engagement of local communities.^42^ It is essential that local stakeholders take ownership of improvement efforts and that the efforts be tailored to local needs and conditions.

An emphasis on understanding local conditions lies at the heart of the work of RAD-AID, an international, nonprofit organization founded in 2008 that aims to expand the availability of medical imaging services in developing countries.^51^ RAD-AID has developed the Radiology-Readiness assessment survey, a tool for evaluating how available resources can best be used to improve imaging in the service of population health in any given locality.^52,53^ It calls for the collection of detailed data on many subjects, including basic local infrastructure (eg, transportation, telecommunications), the energy supply for powering imaging equipment; the prevalence of communicable and noncommunicable diseases, and the availability of drugs, healthcare personnel, diagnostic tests, and medical procedures. It addresses the fact that the value of imaging depends entirely on the context in which it is used. For instance, a mammography program will obviously be of little benefit if there are no surgeons, radiation oncologists, or oncologists available to treat breast cancers, and a donation of equipment will be of no help in a place without electricity or personnel able to use and maintain it properly.^52,53^ The information collected helps determine whether a given intervention is worthwhile or what ancillary components may be needed to make it so. The tool highlights the importance of interdisciplinary collaboration in advancing imaging services.

RAD-AID, which is affiliated with the United Nations and the WHO, aims to foster partnerships between nonprofit organizations, the private sector, government agencies, technology companies, and health care institutions.^54^ The annual RAD-AID conference brings together representatives from these various types of entities to share their ideas and their experiences working to improve imaging services in developing countries. As highlighted in a white paper from the 2010 conference, keys to the long-term success of such efforts include business financing and training for imaging entrepreneurs, the development of information technologies for knowledge transfer, and the development of effective models for providing clinical training and low-cost imaging.^55^

The organization Imaging the World (ITW) has developed one promising model that combines teleradiology with imaging technology that requires limited user training. Specifically, ITW trains local health-care staff to perform so-called volume scanning ultrasound protocols, which require only the use of external landmarks and no knowledge of internal structures; the images can then be sent via cell phone to radiologists with the expertise to interpret them.^55-57^ ITW has garnered the support of major donors, such as the Bill and Melinda Gates Foundation, and has established partnerships with companies from the medical imaging device and informatics industries. Their model has been piloted in Uganda as a means of monitoring maternal and fetal health and has obvious potential applications for cancer care.^57^

Clinical training of staff in low-resource regions can be bolstered by online courses or learning modules, some of which are available on the websites of RAD-AID and other organizations. However, to foster long-term, systemic improvements and the development of local expertise, face-to-face intensive training programs are particularly valuable. Not only do they allow the acquisition of practical, hands-on experience under direct supervision but also they can foster the development of long-term mentoring and strong cross-cultural collaborative relationships. In turn, such relationships can help trainees develop the leadership skills and connections to effect change in their countries of origin, such as the initiation of teaching, research, or cancer screening programs.

One highly successful example of this kind of approach is a teach-the-teachers program run by the Jefferson University Research and Education Institute (JUREI).^55,58^ In the JUREI program, physicians from the developing world undergo training in ultrasound at centers of excellence in the United States and, subsequently, often go on to establish training facilities in their home countries. To date, local training programs in ultrasound have been established at more than 70 JUREI-affiliated centers in 55 countries.^58^ The JUREI program illustrates how, with a relatively small investment of time and resources, it is possible to make a positive impact that will continue to grow over time. Health care institutions across the United States and Europe are involved in international outreach programs that aim to produce a similar long-term effect.

The large international imaging societies, through their existing programs and activities, are uniquely placed to promote scientific exchange and offer education to radiologists from low-resource regions. The European School of Radiology, for example, works with imaging leaders in such countries to identify outstanding candidates for oncologic imaging fellowships in the United States. In addition, the ESR recently took several measures to strengthen its efforts in Latin America, including the provision of 100 subsidized places at the European Congress of Radiology exclusively for Latin American applicants, as well as the opening of an office in Bogota with a full-time staff member to represent the society on site at Latin American radiology congresses. The interest in and need for such initiatives are reflected by the fact that a large proportion of members of the ESR—some 19%—are from Latin America.^59^

Governments of developing nations may also reach out directly to foreign centers of excellence or nonprofit organizations to develop training programs, or they may work with industry to design customized imaging facilities that meet their individual needs. Although there is no one-size-fits-all solution to fulfilling the cancer imaging needs of developing countries, the greater the number of people and disciplines involved in the effort, the faster solutions can be found and implemented. Potential volunteers include not only experienced, fully employed physicians and staff but also trainees and recent retirees, who may have more flexibility. For example, the recently formed International Cancer Experts Corp, a member of the Union for International Cancer Control, seeks to include trainees, early career leaders, senior health care workers, and retirees in multidisciplinary panels of experts assigned to provide training and mentoring in protocol-based cancer care. Working through a designated hub (which may be an academic center, private practice, or professional organization in the developed world), the volunteer experts may be asked to both conduct short initial training visits to health care centers in low-income regions and provide ongoing support through teleconferencing and telecommunications.^60^

International efforts to provide oncologic imaging training and expertise in underserved regions must be accompanied by the political will from the governments of these regions to develop long-term strategies to improve diagnostic facilities and expand the availability of imaging equipment and treatment. Furthermore, to ensure the sustainability of any improvements, measures must be taken to retain trained and specialized oncologic radiologists and oncologists in the public service and give them appropriate resources and structures to train others.

In conclusion, cancer as a major global health care problem is expected to worsen because of a growing and aging population as well as harmful environmental conditions in expanding urban areas, especially in developing countries. The quality of patient care everywhere is affected by the quality of cancer imaging. Dramatic regional disparities in the availability of imaging equipment need to be addressed. Furthermore, there is a shortage of appropriately trained oncologic imagers worldwide. To advance oncologic imaging, training opportunities in both developed and developing countries must be expanded and tailored to regional needs. Large professional societies have increasingly been providing leadership in the creation of clinical practice guidelines and curriculum development as well as offering online and in-person learning opportunities. More such efforts are needed, along with increased advocacy to raise awareness of the importance and complexity of oncologic imaging among the medical community and government policymakers. With respect to improving oncologic imaging in low-resource countries in particular, businesses, nonprofit organizations, and health care institutions have demonstrated that it is possible to establish successful collaborations with local governments and health care organizations either directly or by working through member organizations such as RAD-AID. Tools for telemedicine can be of great help to these efforts, as will developments in artificial intelligence. Nevertheless, the unmet needs for oncologic imaging around the world remain vast. Radiologists at all levels—those in training, midcareer, or even retired—are greatly needed to contribute clinical expertise, teaching skills and mentorship in multidisciplinary efforts to improve cancer care.
